# Plant growth and stress-regulating metabolite response to biochar utilization boost crop traits and soil health

**DOI:** 10.3389/fpls.2023.1271490

**Published:** 2023-10-12

**Authors:** Nyumah Fallah, Ziqin Pang, Zhaoli Lin, Wenxiong Lin, Sylvain Ntambo Mbuya, Ahmad Yusuf Abubakar, Kabore Manegdebwaoga Arthur Fabrice, Hua Zhang

**Affiliations:** ^1^ Key Laboratory of Sugarcane Biology and Genetic Breeding, Ministry of Agriculture, Fujian Agriculture and Forestry University, Fuzhou, China; ^2^ Fujian Provincial Key Laboratory of Agro-ecological Processing and Safety Monitoring, College of Life Sciences, Fujian Agriculture and Forestry University, Fuzhou, China; ^3^ Département de production végétale, Laboratoire de Recherche en Biofortification, Defense et Valorisation des Cultures (BioDev), Faculté des Sciences Agronomiques, Université de Lubumbashi, Lubumbashi, Democratic Republic of Congo; ^4^ Key Laboratory of Crop Ecology and Molecular Physiology, Fujian Agriculture and Forestry University, Fuzhou, China

**Keywords:** biochar, metabolites, crop traits, soil health, soil fertility

## Abstract

**Introduction:**

The utilization of biochar (BC) as a soil amendment in agriculture has gained significant traction among many farmers and researchers, primarily due to its eco-friendly role in boosting crop output. However, the performance of specific metabolites (e.g., zeatin, melatonin, sucrose, and phenyllactic acid) in the various tissues of sugarcane plant (leaf, stem, and root) and rhizosphere soil-deemed plant growth and stress regulators in a long-term BC-amended field remains poorly understood. Additionally, literature on the shift in soil attributes and crop growth triggered by the strong response of these bioactive compounds to longterm BC utilization remains undocumented.

**Methods:**

Metabolome integrated with highthroughput sequencing analyses were conducted to identify and quantify the performance of plant growth and stress-regulating metabolites in a long-term BC-amended field. Additionally, we investigated how the response of these compounds to BC-treated soil influences crop traits and soil biochemical properties.

**Results:**

We also identified and quantified the performance of pathogenic bacteria and unraveled the association between these compounds and potential plant growth-promoting bacteria. The BC-supplemented soil significantly boosted the crop traits, including brix, sucrose content, and chlorophyll, as well as soil nutrients, such as soil total nitrogen (TN), ammonium (NH_4_^+^-N), and nitrate (NO_3_^-^-N). We also noticed that metabolite-deemed plant growth and stress regulators, including melatonin and phenyllactic acid, were enriched considerably in the stem and root tissues of the BC-amended soil. Zeatin in the leaf, stem, and root tissues exhibited the same trend, followed by sucrose in the leaf tissue of the BC-treated soil, implying that the strong response of these compounds to BC utilization contributed to the promotion of crop traits and soil quality. Pathogenic bacteria belonging to Proteobacteria and Acidobacteria were suppressed under the BC-supplemented soil, especially in the root tissue and rhizosphere soil, whereas plant growth-regulating bacteria, mainly *Bradyrhizobium*, responded strongly and positively to several metabolites.

**Discussion:**

Our finding provides valuable information for agronomists, farmers, and environmentalists to make informed decisions about crop production, land use, and soil management practices. Proper soil assessment and understanding of the interaction between the attributes of soil, BC, and metabolites are essential for promoting sustainable agriculture practices and land conservation.

## 1 Introduction

The world’s growing population has necessitated more food production (Van-Dijk et al., 2021). Farmers heavily rely on agriculture inputs, including insecticides, pesticide products, and chemical fertilizers, to boost crop growth and production to meet this challenge (Popp et al., 2013; Pang et al., 2022). However, adopting these agricultural inputs has been associated with several environmental issues, including depletion of soil nutrients, microbial abundance, diversity, and community. Blanchet et al. (2016) documented that soil biological properties (e.g., microbial biomass, phospholipid-derived fatty acid contents, and earthworm biomass and composition) and crop yield performed poorly under nitrogen (N)-treated soil compared with organic amendments soil. Likewise, Luan et al. (2020) found that soil organic carbon (C), available N, phosphorous (P), and microbial communities and functional diversity reduced under N application. Carpenter et al. (1998) reviewed the non-point pollution of surface waters with N and P. They revealed that eutrophication was a widespread problem in lakes, estuaries, rivers, and coastal oceans induced by the over-enrichment with P and N. Hence, farmers are increasingly pressured to safely and sustainably boost agricultural productivity without compromising environmental quality (Bulluck et al., 2002). One alternative to synthetic agriculture inputs is adopting an organic farming system.

Organic farming is a sustainable and holistic agricultural system that highlights the use of natural materials and processes to enhance soil quality, plant health, and ecosystem balance (Watson et al., 2002). Organic soil amendment practices, such as compost, manure, and biochar (BC), have shown great potential in reverting the adverse environmental impacts of synthetic agriculture (Schärer et al., 2022). These substrates play key roles in moderating greenhouse gas emissions, establishing reliable C storage, and promoting environmental functions (Woolf et al., 2010), primarily due to its distinct characteristics (e.g., high porosity, typically alkaline, and large specific surface area) (Tan et al., 2017). Additionally, this substrate can alter a wide range of soil attributes (Abujabhah et al., 2018; Pang et al., 2022), namely, soil pH, soil water-holding capacity, bulk density, soil C and N content, and microbial community. For instance, Ginebra et al. (2022) found that wood BC-treated soil boosted soil total C, reduced soil nitrous oxide (N_2_O) emissions, and promoted soil C storage. Gul et al. (2015) also documented that BC produced from wood and lignocellulosic-rich feedstocks had beneficial effects on soil microbial abundance. Similarly, a recent study revealed that BC-treated soil was crucial in mitigating soil N_2_O emissions in a continuous cropping system (Yang et al., 2022). However, BC being rich in C but low in crucial soil nutrients, such as P, potassium (K), and N (Jindo et al., 2014), could trigger poor soil microbial response and other soil attributes (Ramlow et al., 2019). Wang et al. (2015b) revealed that the reduction in ammonia-oxidizing bacterial abundance reduced N nitrification in an orchard field supplemented with BC. It was recently reported that BC-supplemented soil triggered a decrease in ammonium (NH_4_^+^-N) and nitrate (NO_3_^−^-N) (Martí et al., 2021). Ramlow et al. (2019) also documented that BC application did not influence N availability within the entire soil profile under a broadcast woody BC in Colorado. These discrepancies could be mainly associated with the characteristics of BC, soil composition, and crop types (Liu et al., 2021), which have made it more challenging to precisely estimate the structural effect of BC on vital soil attributes, including soil N and plant metabolites in agriculture soils. As such, it is vital to conduct further studies to comprehensively understand how BC structural effect influences other soil parameters, such as metabolites.

Plant metabolites/bioactive compounds are deemed essential mediators between species, the environment, and agricultural systems (Hartmann, 2007). In the past decades, a large body of research has shed light on the roles of metabolites in plant growth, development, and productivity (Erb and Kliebenstein, 2020; Pang et al., 2021). These compounds also act as antibiotics, photoprotectants, signaling molecules (Mithöfer and Boland, 2012), repellents, and toxins to protect plants against insect pests (Hu et al., 2021). We recently pointed out that the high presence of D-fructose 6-P, D-glucose 6-P, and glucose1-P in sugarcane plant were key in promoting high sugar accumulation (Yuan et al., 2022). In a related study, metabolites, including flavonoids, phenolic, anthocyanins, proanthocyanidins, and carotenoids, were crucial in maintaining and enhancing crop rinds (Junaid Rao et al., 2022). In addition, the excretion of these compounds in soil environments can influence a wide range of soil attributes, particularly in plant rhizosphere zones (Wen et al., 2022). Panchal et al. (2022) mentioned that plant bioactive compounds functioned as a source of soil organic C in forests and grasslands. A related study revealed that the strong response of purine metabolism to the sugarcane/peanut intercropping system improved soil pH, total P, K, available N, acid phosphatase, and urease activities (Tang et al., 2022). However, information regarding the performance of specific metabolites within the various tissues of sugarcane plant (leaves, stems, and roots) and the rhizosphere soil, which are considered regulators of plant growth and stress, in a field subjected to long-term utilization of BC remains poorly understood. Additionally, literature on the shift in soil parameters (e.g., soil biochemical properties and disease-causing bacteria) and crop traits triggered by the strong response of these compounds to long-term BC utilization remains undocumented. Hence, this work aims to (i) illuminate the underpinning mechanism of how the strong response of zeatin, melatonin, sucrose, and phenyllactic acid to BC utilization shapes crop growth and soil biochemical properties and (ii) identify and quantify the performance of pathogenic bacteria and unraveled the association between these compounds and potential plant growth-promoting bacteria.

## 2 Materials and methods

We conducted a field experiment from March 2019 to December 2022 at the Sugarcane Research Center of Fujian Agriculture and Forestry University (26°08′N, 119°23′E) in Cangshan District, Fujian Province, China. The region has an annual average temperature of 20°C, 1,369 mm rainfall, and a subtropical climate. The experiment was conducted in a randomized block design consisting of two treatments: biochar applied at the rate of 20 t ha^−1^ (BC) and control (CK). Each treatment contained three replicates, covering an area of 25 m^2^. Sugarcane stalks were cut approximately 10–12 cm in length, maintaining two buds on each set. Subsequently, 10 sets were planted in each row, ensuring a spacing of 0.3 m between plants and 0.5 m between rows (Fallah et al., 2021). We supplemented the BC and CK treatments with 375 kg/hm^2^ of compound fertilizer (N-P_2_O_5_-K_2_O 15-15-15) in April 2019. The BC was made from sugarcane straw, using fast pyrolysis (550−650°C). The sugarcane variety “ROC22” was adopted as planting material. It is a common sugarcane variety in Fujian Province, China. It possesses high yield, high sugar content, drought resistance, strong tillering ability, a high stemming rate, excellent growth, cold resistance, remarkable adaptability, and disease hardiness. Additional information regarding the study site is mentioned in **Table 1**.

**Table 1:** Initial characteristics of the soils and biochar.

Parameters	Soil properties	Biochar properties
Total carbon (TC), kg^−1^	7.22	35.08
Total nitrogen (TN), kg^−1^	3.54	2.09
Potential of hydrogen (pH)	8.89	10.11
Available potassium (AK), kg^−1^	9.01	13.66
Available phosphorous (AP), kg^−1^	13.11	7.07
Organic matter (OM), kg^−1^	16.22	7.82
Electrical conductivity (EC), dS m^−1^	3.11	3.2
Carbon/nitrogen ration (C/N)	2.03	16.78

### 2.1 Sampling and preparation of rhizosphere soil and plant tissues

Sampling of the rhizosphere soil and various plant tissues, including leaf, stem, and root, was conducted in December 2022 using the methods we adopted in a previous study (Fallah et al., 2023a). In summary, random sampling of plant tissues of three healthy sugarcane plants was carried out from each plot as a biological replicate. The plant tissues were washed using phosphate buffer solution and cleaned using 75% alcohol cotton. We wrapped the samples in tin foil, placed them in liquid nitrogen for 5 min, stored in dry ice, transported to the laboratory, and stored at −80°C. The sample of soil taken from the plant root zones was classified as rhizosphere soil, with each group of the soil sample containing three replicates. Finally, we generated a total of 36 samples. Later, we air-dried a portion of the soil sample, which was ground and sieved using a 2-mm mesh to investigate soil chemical properties.

### 2.2 Assessment of sugarcane traits and soil chemical properties

Extech Portable Sucrose Brix Refractometer (Mid-State Instruments, CA, United States) was used to estimate sucrose content and measured using the formula: sucrose (%) = Brix (%) × 1.0825–7.703 (Yuan et al., 2022). A random sampling of 25 sugarcanes was conducted in each row to estimate the diameter of the plants using a vernier. Sugarcane heights were measured in centimeters (cm) from the soil’s surface to the sugarcane’s top by randomly sampling 25 plants in each row using a meter rod. We added the average of three replicates to determine the mean of sugarcane heights.

An element analyzer (vario MAX cube, Germany) was used to estimate the TN. Soil potential of hydrogen (pH) was tested using a glass electrode pH meter. We used fresh soil samples to extract soil NO_3_^−^-N and NH_4_^+^-N with 2.0 M KCl and calculated using a continuous-flow analyzer (San++, Skalar, Holland) (Zhao et al., 2019).

The soil samples were incubated using buffer sodium carboxymethylcellulose solution and cellulose activity (glucose, mg/g 24 h, 37°C) to assess colorimetrically by measuring a decrease in 3,5-dinitrosalicylic acid from reducing sugar. We estimated soil urea activity (NH_3_-N, mg/g 24 h, 37°C) using sodium hypochlorite colorimetry and improved sodium phenolate. Acid phosphatase activity was tested using a nitrophenyl phosphate disodium substrate (phenol, μg/g, 1 h, 37°C). Soil β-glucosidase activity was measured with a colorimetric p-nitrophenol assay after buffering the soil with p-nitrophenyl-β-glucopyranoside (p-nitrophenyl, μg/g, 1 h, 37°C). The measurement of soil urease activity involved incubating the samples with 20 mL of citric acid buffer (pH 6.7) and 10 mL of 10% urea solution at 37°C (24 h) to assess the release of NH_4_^+^-N (Sun et al., 2014).

### 2.3 Metabolite extraction, processing, and annotation

We conducted metabolite extractions following the methods documented in previous works (Chen et al., 2013; Chenkun et al., 2021). The various samples were ground into powder at 30 Hz using a mixer mill (MM400, Retsch) for 1.5 min after freeze-drying. We used 100 mg powder to conduct metabolite extraction. The extraction was performed at night at 4°C using 0.8 mL aqueous methanol (methanol: H_2_O_2_, 70:30, v/v) and pure methanol and subsequently centrifuged for 10 min at 10,000 g. We collected, homogenized, and filtered the supernatants (SCAA-104, 0.22 mm pore size; ANPEL Shanghai, China, www.anpel.com.cn/). Detailed information regarding further processing and annotation of the samples was documented in recent studies (Fallah et al., 2022).

### 2.4 Extraction of DNA, PCR amplification, sequencing, and data processing

The extraction of genomic DNA was performed by employing the Fast DNA™ Spin Kit (MP Biomedicals, LLC, Santa Ana, USA) using 0.5 g fresh soil as recommended by the manufacturer. DNA absorbance (A260 and 280 nm) was computed using BioTek Synergy H1 Hybrid Multi-Mode Microplate Reader (BioTek, USA) to investigate the quantity and quality of DNA. The bacterial 16S rRNA gene hypervariable V3–V4 region was amplified using 341F and 805R primers. PCRs were conducted in a 50-µL combination using 1 mM dNTPs (deoxynucleoside triphosphate), 1 × PCR buffer, 1 U of Platinum Taq, DNA template (10 ng), and each primer at 5 µM. PCR amplifications with an initial denaturation at 94°C (3 min), denaturation (five cycles at 94°C, 30 s), annealing at 45°C (20 s), and extension at 65°C (30 s) were performed. We subsequently carried out denaturation (20 cycles at 94°C) (20 s), annealing at 55°C (20 s), extension at 72°C (30 s), and the last extension at 72°C (5 min). Finally, an Illumina HiSeq 2500 platform (2 × 250 paired ends) at Biomarker Technologies Corporation, Beijing, China, was employed to conduct high-throughput sequencing. The raw data were later deposited on the NCBI Sequence Read Archive platform (accession no. PRJNA929962).

FLASH was adopted to merge the paired-end reads of the original DNA fragments (Tan et al., 2017). The paired-end reads appropriated to each sample were aligned to a sample-specific barcode. Sequence clusterings were conducted with operational taxonomic units (OTUs) based on 97% similarity. Thereafter, taxonomic information was annotated for each sequence using the Ribosomal Database Project (RDP) (Wang et al., 2007). Sequences that contained low quality and did not correspond with the barcode or primer with a high average quality score (Q ≥ 20) or without ambiguous base pairs were excluded. We then clustered all the sequences at 97% nucleotide similarity. Taxonomic classification was carried out using the SILVA database (SILVA Release 138, Bacteria), and biomarker biocloud platform (www.biocloud.net) was used to conduct the bioinformatics analysis.

### 2.5 Statistical analysis

We employed Bioconductor (http://www.bioconductor.org/) package “Mfuzz” and R software (http://www.r-project.org/) to investigate the expression patterns of metabolites based on fuzzy c-means. We adjusted the fuzzification parameter to m = 2 and the number of clusters to c = 12 to retain the soft clustering of the entire metabolite. Ternary plot analysis was conducted using R language-based packages, namely, grid and ggtern, an extension of the package ggplot2 to identify the upregulated and downregulated metabolites in the different compartments. Interactive networks of plant–soil systems and metabolites were conducted to establish the association between metabolites in the different compartments and under both treatments (Toju et al., 2016). The correlations between metabolites and plant growth-promoting bacteria were assessed using a correlation matrix. The potential pairwise Spearman’s ranks were determined and displayed using Cytoscape (version 3.6.1). ANOVA was used to evaluate the test data and visualized with GraphPad Prism (version 10.0.0). Tukey’s HSD test (*p* < 0.05) was used to compare the difference between mean values. The Biomarker biocloud platform (www.biocloud.net) was used to generate the rest of the used in this work.

## 3 Results

### 3.1 Sugarcane traits and soil chemical properties respond to biochar-supplemented soil

**Figure 1:**
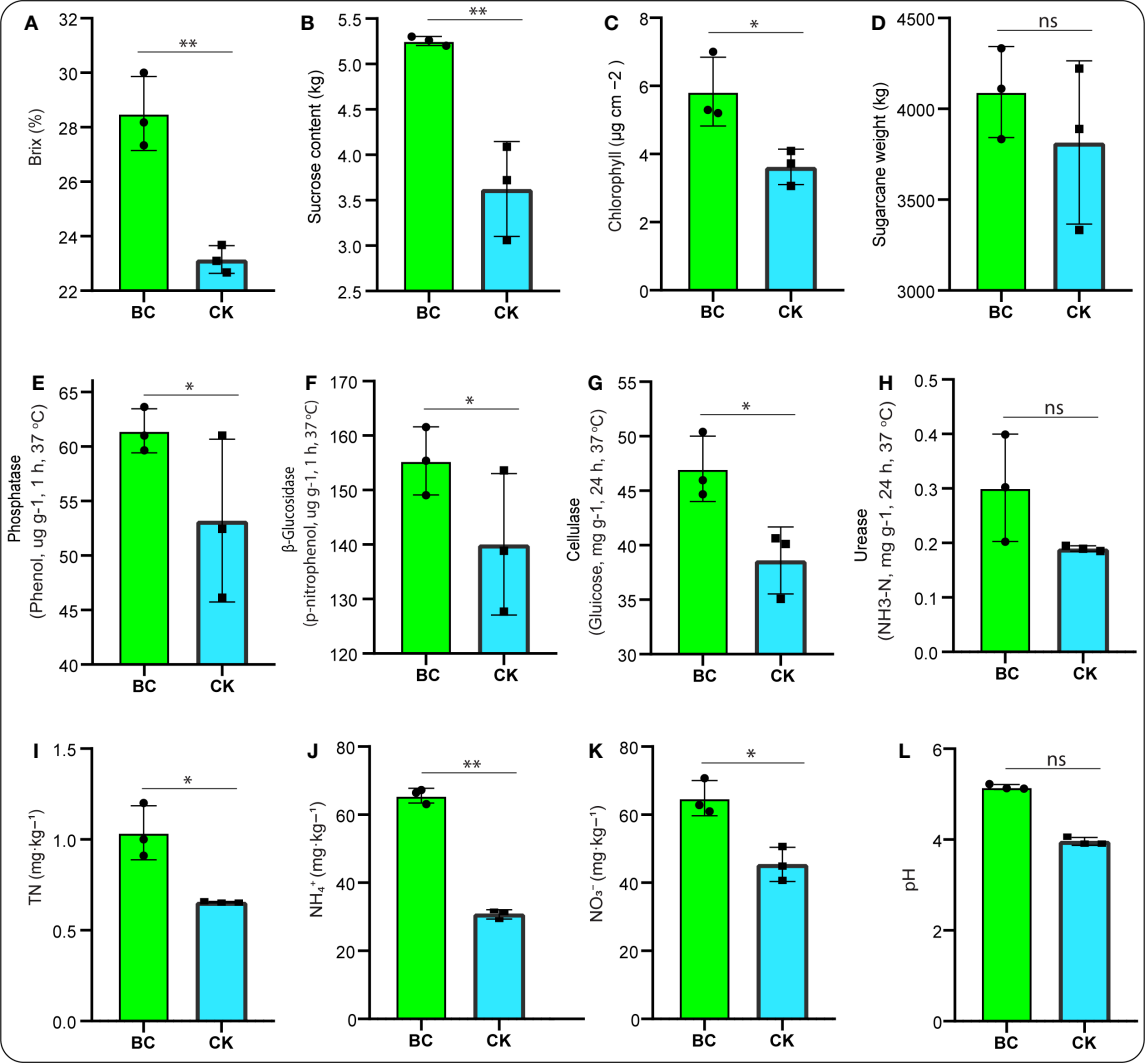
Bar graphs illuminating crop traits **(A–D)**. Soil enzyme activities: **(E–H)**, followed by soil TN, total nitrogen **(I)**; soil NH_4_^+^-N, ammonium **(J)**; NO_3_^−^-N, nitrate **(K)**; and pH, potential hydrogen **(L)**. Graph with asterisk mark depicts significant differences between treatments, while “ns” stands for not significant (Tukey test, *p* < 0.05).

Sugarcane traits responded strongly to the BC-supplemented soil. For instance, the crop brix, sucrose content, and chlorophyll content significantly increased (*p* < 0.05) under the BC-supplemented soil compared with the CK treatment (**Figures 1A–C**). Additionally, **Figure 1D** showed that the crop weight was promoted under the BC-supplemented soil, but no significant difference was observed between both treatments.

Meanwhile, we also noticed that soil chemical properties, including phosphatase, β-glucosidase, and cellulase, peaked considerably (*p* < 0.05) under the BC-supplemented soil than the CK treatment (**Figures 1E–G**). In addition, soil urease activity under the BC-supplemented soil outperformed those in the CK treatment but revealed no significant difference (**Figure 1H**). **Figures 1I–K** demonstrates that soil NH_4_^+^-N, followed by TN and NO_3_^−^N, were significantly increased (*p* < 0.05) under the BC-supplemented soil relative to the CK treatment. In addition, soil pH exhibited a 55.01% increase under the BC-supplemented soil compared with a 44.99% increase under the CK treatment (**Figure 1L**).

### 3.2 Metabolite composition and relative abundance in the different compartments under the different treatments

**Figure 2:**
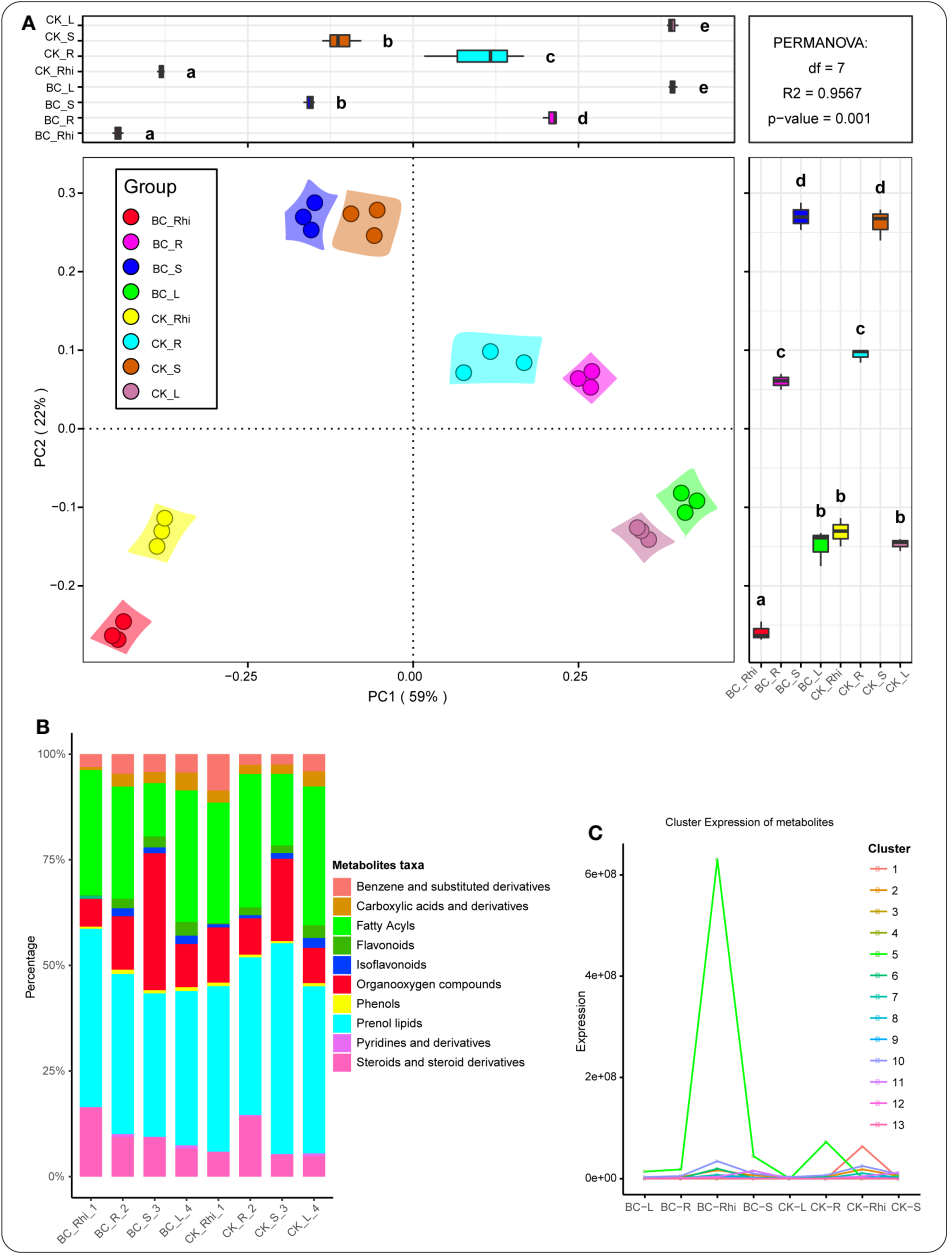
Principal component analysis (PCoA) of the entire metabolites in the various samples **(A)**. Relative abundance of metabolites detected in the entire compartments under the different treatments **(B)**. K-means clustering trend chart displaying the decrease and increase trends of metabolites under both treatments in the various plant compartments. The color lines in the graph symbolize the average change trend of metabolite in each k-means cluster between groups **(C)**. BC_Rhi, rhizosphere soil of the biochar-supplemented soil; BC_R, root tissue of the biochar-supplemented soil; BC_S, stem tissue of the biochar-amended soil; BC_L, leaf tissue of the biochar-supplemented soil; followed by CK_R, control root tissue; CK_Rhi, control rhizosphere soil; CK_S, control stem tissue; and CK_L, control leaf tissue. Different lowercase letters signify the various groups or categories of samples.

We conducted principal coordinate analysis (PCoA) to assess metabolite composition in the various compartments under both treatments. The metabolite composition distribution trend was largely compartment-driven (**Figure 2A**). The relative abundance of metabolite taxa in the different compartments, including prenol lipids (31.42%), fatty acyls (21.12%), organooxygen compounds (10.78%), steroids and steroid derivatives (8.73%), and benzene and substituted derivatives (3.53%), was dominant. Furthermore, a number of taxa, including carboxylic acids and derivatives (1.61%), flavonoids (1.89%), isoflavonoids (0.83%), phenols (0.52%), pyridines and derivatives (0.14%), and other (18.44%) were more prevalent (**Figure 2B**). **Figure 2C** reveals that these aforementioned taxa were more pronounced in the rhizosphere soil of the BC-supplemented soil, followed by the rhizosphere soil of the KC treatment.

### 3.3 Expression pattern of metabolites detected in the various treatments in the different plant compartments

**Figure S1** further indicates that the distribution pattern of metabolite composition was primarily compartment-driven, suggesting that metabolite composition is more sensitive to different plant compartments rather than fertilization.

**Figure 3:**
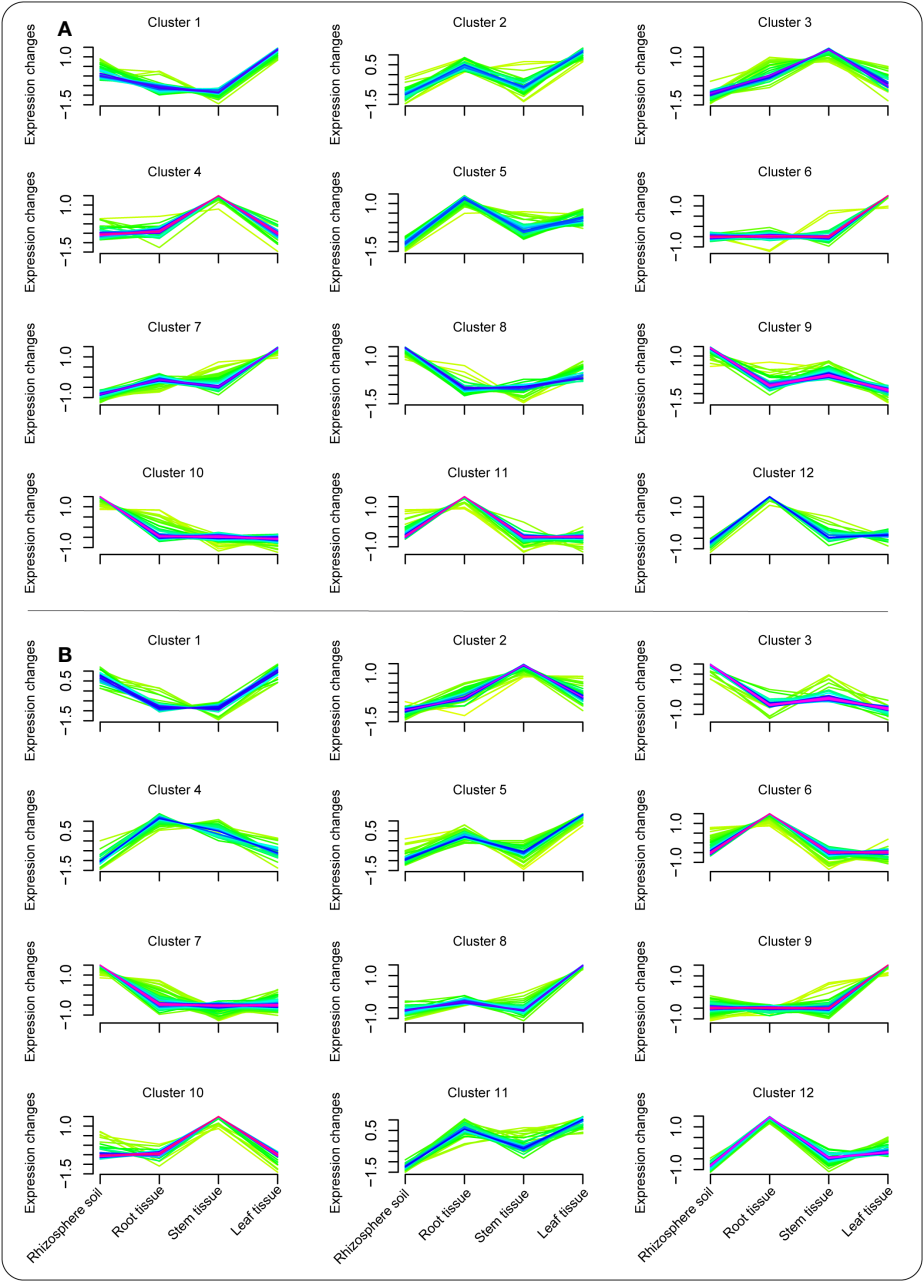
Cluster analysis revealing the expression pattern of metabolites in the different plant tissues/compartments under the BC-supplemented soil **(A)** and the CK treatment **(B)**.

Mfuzz package was later used to explore the expression pattern of metabolite composition in each compartment under the BC-supplemented soil (**Figure 3A**) and the CK treatment (**Figure 3B**). The results showed that a number of essential metabolites peaked in the different clusters. In the BC-supplemented soil, jasmonic acid was among many metabolites that peaked in the leaf tissue in cluster 1. Moreover, benzaldehyde and benzene were some of the metabolites demonstrating a similar trend in cluster 2 in the root and leaf tissues. Similarly, phenylacetic acid and fluridone in cluster 3 peaked considerably in the stem tissue, whereas tyramine, homoveratric acid, and sebacic acid were considerably high in the stem and root tissues of clusters 4 and 5, respectively. In addition, mesaconate, biocytin, and abscisic acid were some of the dominant metabolites that peaked in the leaf tissue in clusters 6 and 7, respectively. Abscisic aldehyde, acetoin, and traumatic acid were among the dominant metabolites detected in rhizosphere soil in clusters 8, 9, and 10, respectively. Apigenin exhibited a similar pattern in the root and leaf tissues in cluster 11, whereas paromomycin increased significantly in cluster 12, especially in the root tissue (**Figure 3A**; **Table S1**).

In the CK treatment, apigenin and jasmonic acid were among the metabolites that peaked in both rhizosphere soil and leaf tissue in cluster 1. Ononin and pogostone revealed a similar trend in the stem and rhizosphere soil in clusters 2 and 3, respectively. Tyramine and daidzein were some of the metabolites that were considerably expressed in the root tissue in clusters 4 and 6, whereas pisatin, succinic acid semialdehyde, and retinal exhibited a similar pattern in the leaf tissue in clusters 5, 8, and 9, respectively. Likewise, coumestrol was considerably expressed in the rhizosphere soil in cluster 7, whereas sucrose and betanin in clusters 10 and 12 peaked in stem and root tissues, respectively. Abscisic acid demonstrated a similar trend in both root and leaf tissues (**Figure 3B**; **Table S2**).

### 3.4 Differentially upregulated and downregulated metabolites in the different compartments of the crop

**Figure 4:**
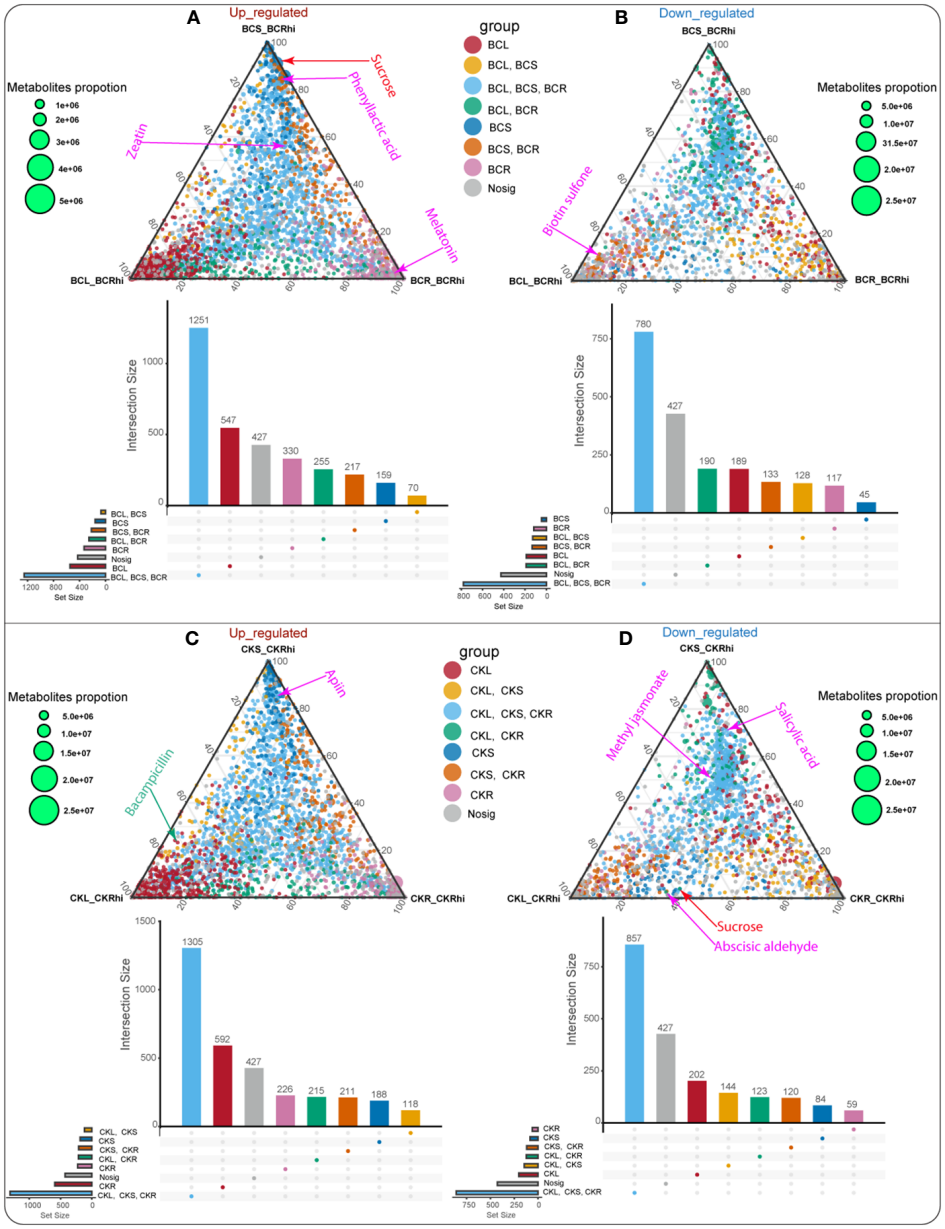
Ternary plot of the entire metabolites identified in the leaf tissue (BCL_BCRhi), stem tissue (BCS_BCRhi), and root tissue (BCR_BCRhi). Upregulated metabolites identified in the BC-supplemented soil and the CK treatment **(A, C)**, and downregulated metabolites detected in the BC-supplemented soil and the CK treatment **(B, D)**. Each circle signifies the downregulated and upregulated metabolites. Its position indicates its relative abundance in the leaf, stem, root tissues, and rhizosphere soil, and its size symbolizes the average in the compartments. The upregulated or downregulated metabolite in one compartment relative to the other is characterized by the colored circle. Red circles signify the specific metabolite in the leaf tissue. Yellow circles indicate the overlapping metabolite in the leaf and stem tissues. Light blue circles depict the overlapping metabolite detected in the leaf, stem, and root tissues. Green circles symbolize the overlapping metabolite in the leaf and root tissues. Dark blue circles indicate the specific metabolite detected in the stem tissue. Orange circles signify the overlapping metabolite identified in the stem and root tissues, whereas pink circles denote the specific metabolite detected in the root tissue.

Ternary plot analysis was performed to identify the specific metabolites that were upregulated or downregulated in the different compartments in each treatment (**Figures 4A, B**; **Tables S3**, **4**). The analysis revealed that a number of key metabolites were significantly upregulated (*p* < 0.05) in the different compartments of the BC-supplemented soil, including sucrose and melatonin in the stem and root tissues, respectively. Similarly, phenyllactic acid was considerably upregulated (*p* < 0.05) in the stem and root tissues whereas zeatin exhibited a similar trend in the leaf, stem, and root tissues (**Figure 4A**; **Table S3**). On the other hand, biotin sulfone revealed the opposite in the leaf tissue (**Figure 4B**; **Table S4**).

In the CK treatment, bacampicillin was significantly upregulated (*p* < 0.05) in the stem tissue, whereas apiin revealed a similar pattern in the leaf, stem, and root tissues (**Figure 4C**; **Table S5**). However, some essential metabolites, namely, methyl jasmonate and salicylic acid, were downregulated in the leaf, stem, and root tissues of the CK treatment. Abscisic aldehyde and sucrose demonstrated the same trend in leaf and root tissues (**Figure 4D**; **Table S6**).

### 3.5 Differentially abundant, enriched pathways, and annotated metabolites in the different compartments of the crop

The differential abundance of metabolites showed that steroid biosynthesis, anthocyanin biosynthesis, and monoterpenoid biosynthesis performed better in the BC rhizosphere soil compared with the CK rhizosphere soil (**Figure S2A**). Similarly, the BC rhizosphere soil promoted sphingolipid metabolism, betalain biosynthesis, and glycerophospholipid metabolism compared with the CK rhizosphere soil (**Figure S2B**). Moreover, benzoxazinoid biosynthesis was significantly higher in the leaf tissue of the BC amendment compared with the leaf tissue of the CK treatment (**Figure S2C**). At the same time, sesquiterpenoid and triterpenoid biosynthesis and cutin suberin and wax biosynthesis marked a significant increase in the stem tissue of the BC amendment compared with the stem tissue of the CK treatment (**Figure S2D**).

**Figure 5:**
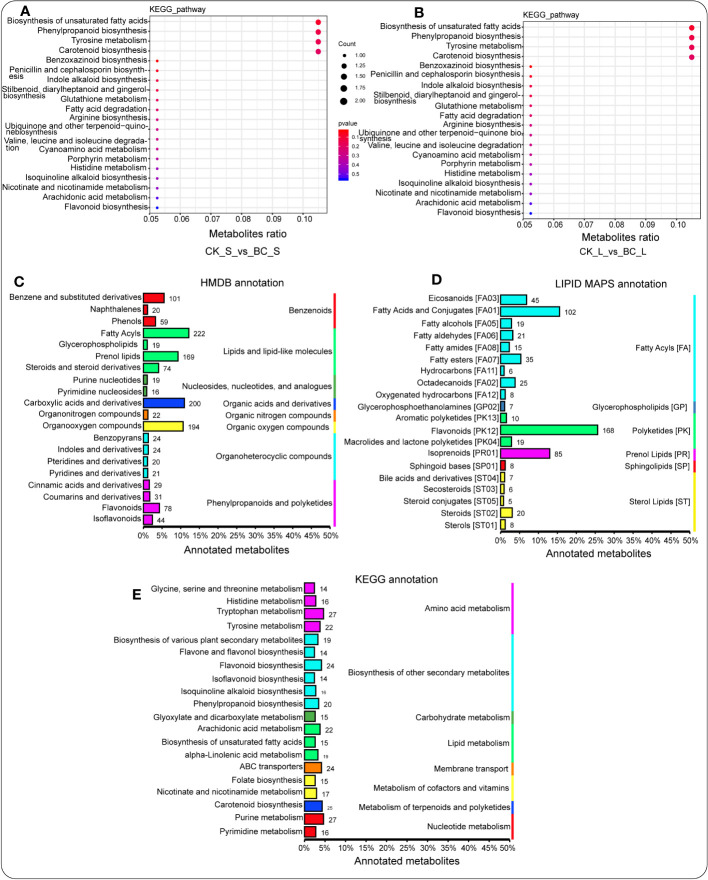
Enriched KEGG signaling pathway of metabolites in the stem tissue **(A)** and the leaf tissue **(B)** under the BC-supplemented field compared with the CK treatment. HMDB database annotations display the hierarchy classification matching the superclass and class information of the HMDB database **(C)**. Lipid maps illuminate the annotated metabolites. The column length characterizes the number of metabolites annotated to a specific classification **(D)**. The column length represents the metabolites annotated. Metabolite annotation using the KEGG pathway. Entry under the same box in the figure symbolizes the hierarchical classification notes of the KEGG pathway. The column length represents the number of metabolites annotated **(E)**.

The enriched KEGG pathway revealed that carotenoid biosynthesis, biosynthesis of unsaturated fatty acids, and alpha-linolenic acid metabolism were enriched considerably in the BC rhizosphere soil compared with the CK rhizosphere soil, followed by arginine biosynthesis, limonene and pinene degradation, and histidine metabolism (**Figure S2E**). In the root tissue of the BC amendment, a number of enriched KEGG pathways, including histidine metabolism; cysteine and methionine metabolism; glycine, serine, and threonine metabolism; betalain biosynthesis; glycerophospholipid metabolism; pantothenate and CoA biosynthesis; aminoacyl-tRNA biosynthesis; and beta-alanine metabolism outperformed those detected in the root tissue of CK treatment (**Figure S2F**). Additionally, the stem tissue of the BC amendment exhibited the advantage of significantly enriching biosynthesis of unsaturated fatty acids, phenylpropanoid biosynthesis, tyrosine metabolism, and carotenoid biosynthesis relative to those detected in the stem tissue of the CK treatment (**Figure 5A**). The leaf tissue of the BC amendment had a similar effect on biosynthesis of unsaturated fatty acids, phenylpropanoid biosynthesis, tyrosine metabolism, and carotenoid biosynthesis relative to those detected in the leaf tissue of the CK treatment (**Figure 5B**).

The pathways of the annotated metabolites of the Human Metabolome Database (HMDB) showed that lipids and lipid-like molecules (e.g., fatty acyls and prenol lipids), organic acids and derivatives (e.g., carboxylic acids and derivatives), and organic oxygen compounds (e.g., organooxygen compounds) were enriched considerably (**Figure 5C**). In addition, LIPID MAPS of metabolite annotation demonstrated that fatty acyls (e.g., fatty acids and conjugates, eicosanoids, and fatty esters), polyketides (e.g., flavonoids), and prenol lipids (e.g., isoprenoids) were considerably high (**Figure 5D**), whereas the KEGG pathway of metabolite annotation revealed the opposite trend (**Figure 5E**).

### 3.6 Bacteria abundance and community in plant compartments response to biochar-supplemented soil

Meanwhile, we also identified a number of bacteria in different plant compartments. The various plant tissues were dominantly occupied by Proteobacteria, Firmicutes, Bacteroidota, Myxococcota, Actinobacteriota, Acidobacteriota, Gemmatimonadota, Nitrospirota, and Patescibacteria (**Figure S3A**). **Figure S3B** reveals that the community composition of these bacteria was region-specific. For example, bacteria identified in the leaf and stem tissues (aboveground compartments) were separated from those in the root tissue and rhizosphere soil (belowground compartments). Later, we employed BugBase functional analysis to confirm the suppressive effect of BC on pathogenic bacteria in the various compartments. We observed that pathogenic bacteria belonging to Proteobacteria were suppressed under the BC-supplemented soil relative to those in the CK treatment, especially in the rhizosphere soil and root tissue. Moreover, pathogenic bacteria belonging to Acidobacteria and Nitrospirae in the rhizosphere soil of the BC-supplemented soil were suppressed relative to those detected in the CK rhizosphere soil. Disease-causing bacteria belonging to Bacteroidetes exhibited similar behavior in the leaf tissue of the plant, whereas those belonging to Firmicutes were also identified in the leaf and stem tissues of the CK plants, implying that the BC-supplemented soil was effective in eradicating pathogenic bacteria belonging to Firmicutes (**Figure S3C**).

### 3.7 Metabolite associations with classified plant growth-promoting bacteria and soil biochemical properties

**Figure 6:**
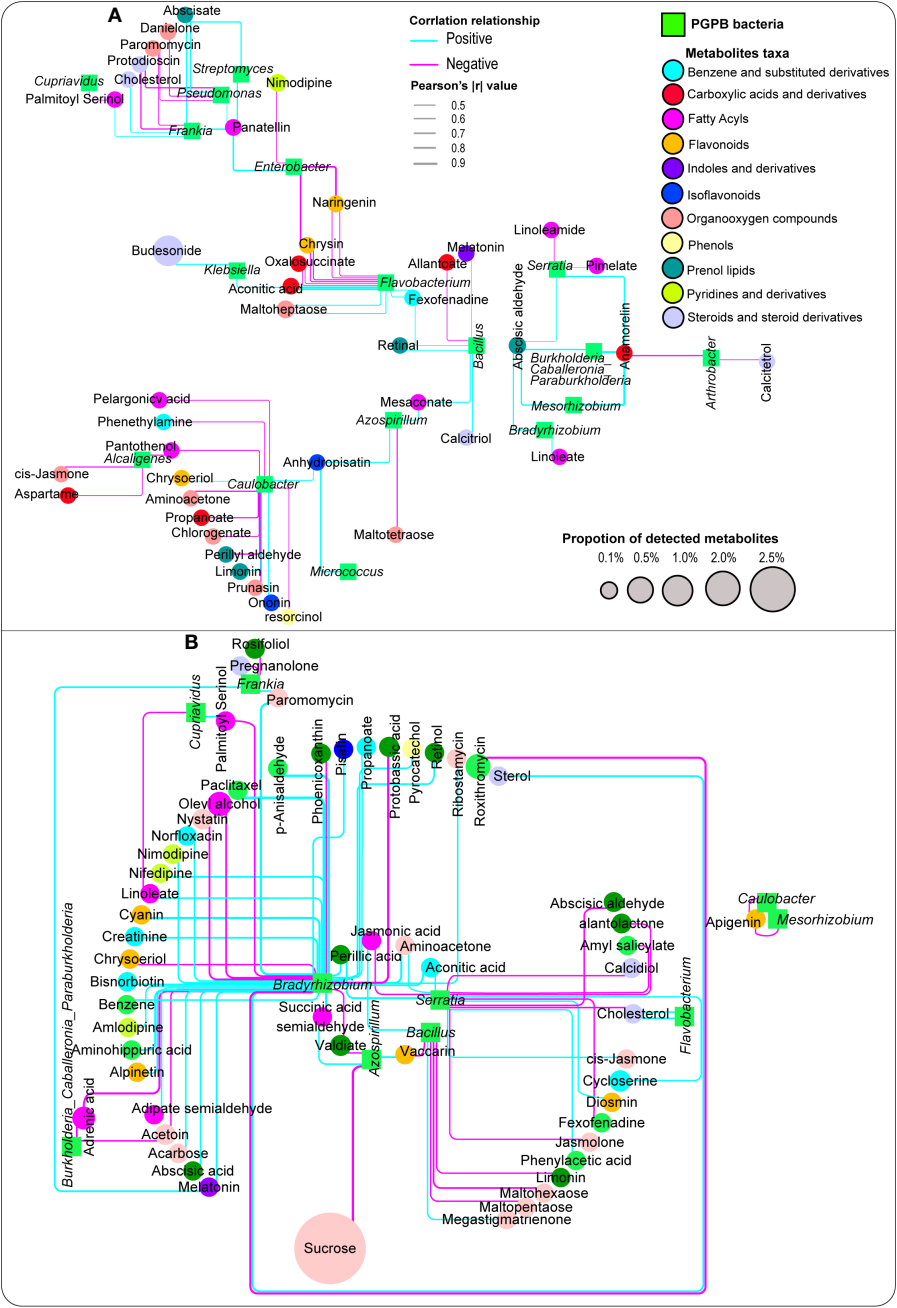
Network analysis depicting the association between metabolites and plant growth-promoting bacteria detected in the aboveground plant tissues (leaf, stem), **(A)** and the belowground compartments (root, rhizosphere soil), **(B)** Pink and blue lines depict negative and positive correlations, respectively.

Metabolite associations with a number of classified plant growth-promoting bacteria were performed, and the result indicated that these targeted bacteria exhibited distinct relationships with a number of vital plant metabolites belonging to different taxa. Noticeably, *Bacillus* had a strong positive correlation with melatonin, whereas *Bradyrhizobium*, *Mesorhizobium*, and *Serratia* were strongly and positively correlated with abscisic aldehyde, whereas *Flavobacterium* displayed a significant positive association with fexofenadine in the aboveground compartments (stem and leaf tissues) (**Figure 6A**; **Table S7**).

In the belowground compartments (root tissue and rhizosphere soil), a significant number of metabolites exhibited strong positive associations with plant growth-promoting bacteria, especially *Bradyrhizobium*. For instance, some important metabolites, including melatonin and abscisic acid, responded strongly and positively to *Bradyrhizobium*, whereas *Enterobacter* showed a significant positive relationship with phenyllactic acid and *Serratia* had a similar pattern with zeatin. Likewise, *Enterobacter* and *Frankia* were significantly and positively correlated with phenyllactic acid and melatonin, respectively (**Figure 6B**; **Table S8**).

We also evaluated metabolite association with soil chemical properties. The results showed that some key metabolites exhibited a significant positive association with soil chemical properties. For example, sucrose, apigenin, and melatonin tended to favor soil TN. Moreover, phenyllactic acid and zeatin exhibited a significant positive association with soil β-glucosidase. Abscisic acid and jasmonic acid were significantly and positively correlated with soil phosphatase. Zeatin favored soil NH_4_^+^-N and β-glucosidase, whereas apigenin and melatonin responded strongly and positively to soil TN (**Table 2**).

**Table 2:** Metabolites identified in the rhizosphere soil correlate with soil biochemical properties.

raw.r	raw.p	Metabolites	Soil properties
0.94	0	Sucrose	TN
−0.83	0.01	Jasmonic acid	Urease
−0.83	0.01	Sucrose	Urease
−0.83	0.01	Abscisic aldehyde	NH_4_^+^-N
0.78	0.02	Phenyllactic acid	β-Glucosidase
0.77	0.02	Abscisic acid	Phosphatase
0.77	0.02	Jasmonic acid	Phosphatase
0.77	0.02	Zeatin	NH_4_^+^-N
0.71	0.03	Apigenin	TN
0.66	0.03	Melatonin	TN
−0.6	0.04	Abscisic aldehyde	TN
−0.6	0.04	Abscisic aldehyde	NO_3_^–^N
−0.6	0.04	Sucrose	NH_4_^+^-N
−0.54	0.05	Abscisic aldehyde	Urease
−0.54	0.05	Apigenin	Urease
−0.54	0.05	Abscisic aldehyde	Cellulase
−0.54	0.05	Apigenin	Cellulase
−0.54	0.05	Sucrose	Cellulase
0.49	0.06	Zeatin	β-Glucosidase
−0.49	0.06	Biotin sulfone	NH_4_^+^-N

Meanwhile, we also established the association between metabolites identified in the various plant tissues with the crop traits (**Tables 3**–**6**). We observed that a number of plant growth and stress-regulating metabolites detected in the leaf tissue demonstrated a significant positive correlation with various crop traits. For example, metabolites, including melatonin, abscisate, and sucrose, showed a significant positive association with the crop brix. Likewise, abscisic acid and melatonin exhibited the same pattern with the crop height and chlorophyll content, respectively (**Table 3**). Metabolites identified in the stem tissues, including abscisic aldehyde, biotin sulfone, and jasmonic acid, showed a significant positive correlation with the crop brix. Additionally, abscisic acid and jasmonic acid were significantly and positively associated with the crop weight whereas biotin sulfone revealed a similar pattern with the crop chlorophyll content (**Table 4**). In the root tissue, metabolites such as abscisate and biotin sulfone revealed a strong and positive association with the crop brix. In addition, abscisate, apigenin, and biotin sulfone demonstrated the same trend with the crop sucrose content (**Table 5**). However, metabolites identified in the rhizosphere soil of the crop revealed no significant association with the traits (**Table 6**).

**Table 3:** Metabolites identified in the leaf tissue correlate with different crop traits.

raw.r	raw.p	Metabolites	Crop traits	Tissue/compartment
0.94	0	Melatonin	Brix	Leaf
0.83	0.04	Abscisic acid	Sugarcane weight	Leaf
0.83	0.04	Abscisate	Brix	Leaf
−0.77	0.05	Phenyllactic acid	Brix	Leaf
0.77	0.05	Sucrose	Brix	Leaf
0.71	0.05	Melatonin	Chlorophyll	Leaf
0.71	0.11	Abscisic acid	Brix	Leaf
−0.66	0.16	Phenyllactic acid	Chlorophyll	Leaf
0.66	0.16	Sucrose	Chlorophyll	Leaf
0.66	0.16	Melatonin	Sugarcane weight	Leaf
0.6	0.21	Abscisate	Chlorophyll	Leaf
0.6	0.21	Abscisate	Sugarcane weight	Leaf
0.6	0.21	Apigenin	Sugarcane weight	Leaf
0.6	0.21	Zeatin	Sugarcane weight	Leaf
0.54	0.27	Abscisic acid	Chlorophyll	Leaf
0.54	0.27	Melatonin	Sucrose content	Leaf
−0.54	0.27	Phenyllactic acid	Sucrose content	Leaf
0.54	0.27	Sucrose	Sucrose content	Leaf
−0.49	0.33	Phenyllactic acid	Sugarcane weight	Leaf
0.49	0.33	Sucrose	Sugarcane weight	Leaf
−0.49	0.33	Abscisic aldehyde	Brix	Leaf
0.43	0.4	Abscisate	Sucrose content	Leaf
-0.37	0.47	Abscisic aldehyde	Chlorophyll	Leaf
0.37	0.47	Abscisic acid	Sucrose content	Leaf
−0.37	0.47	Biotin sulfone	Sucrose content	Leaf
0.37	0.47	Biotin sulfone	Sugarcane weight	Leaf
−0.37	0.47	Biotin sulfone	Brix	Leaf
0.37	0.47	Jasmonic acid	Brix	Leaf

**Table 4:** Metabolites identified in the stem tissue correlate with different crop traits.

raw.r	raw.p	Metabolites	Crop traits	Tissue/compartment
0.94	0	Biotin sulfone	Brix	Stem
0.77	0.01	Biotin sulfone	Chlorophyll	Stem
0.77	0.02	Abscisic acid	Sugarcane weight	Stem
0.77	0.03	Jasmonic acid	Sugarcane weight	Stem
0.77	0.03	Abscisic aldehyde	Brix	Stem
0.71	0.11	Jasmonic acid	Brix	Stem
−0.66	0.16	Abscisic aldehyde	Sucrose content	Stem
0.66	0.16	Biotin sulfone	Sucrose content	Stem
0.66	0.16	Melatonin	Sugarcane weight	Stem
−0.6	0.21	Abscisic aldehyde	Chlorophyll	Stem
0.6	0.21	Zeatin	Sucrose content	Stem
0.54	0.27	Zeatin	Chlorophyll	Stem
−0.54	0.27	Apigenin	Sugarcane weight	Stem
0.54	0.27	Sucrose	Brix	Stem
0.49	0.33	Jasmonic acid	Chlorophyll	Stem
−0.49	0.33	Abscisate	Sucrose content	Stem
0.49	0.33	Abscisic acid	Brix	Stem
0.43	0.4	Zeatin	Brix	Stem
−0.37	0.47	Abscisate	Chlorophyll	Stem
0.37	0.47	Abscisic acid	Chlorophyll	Stem
0.37	0.47	Biotin sulfone	Sugarcane weight	Stem

**Table 5:** Metabolites identified in the root tissue correlate with different crop traits.

raw.r	raw.p	Metabolites	Crop traits	Tissue/compartment
0.83	0.04	Abscisate	Brix	Root
0.83	0.04	Biotin sulfone	Brix	Root
0.77	0.05	Abscisate	Sucrose content	Root
0.77	0.05	Apigenin	Sucrose content	Root
0.77	0.05	Biotin sulfone	Sucrose content	Root
0.71	0.11	Abscisate	Chlorophyll	Root
0.71	0.11	Apigenin	Chlorophyll	Root
0.71	0.11	Biotin sulfone	Chlorophyll	Root
0.66	0.16	Abscisic aldehyde	Chlorophyll	Root
0.66	0.16	Abscisic aldehyde	Sugarcane weight	Root
0.66	0.16	Abscisic aldehyde	Brix	Root
0.6	0.21	Jasmonic acid	Brix	Root
0.54	0.27	Abscisic aldehyde	Sucrose content	Root
0.54	0.27	Apigenin	Brix	Root
0.49	0.33	Phenyllactic acid	Sucrose content	Root
−0.49	0.33	Zeatin	Sugarcane weight	Root
0.43	0.4	Phenyllactic acid	Chlorophyll	Root
0.43	0.4	Sucrose	Chlorophyll	Root
−0.37	0.47	Zeatin	Brix	Root

**Table 6:** Metabolites identified in the rhizosphere soil correlate with different crop traits.

raw.r	raw.p	Metabolites	Crop traits	Tissue/compartment
−0.6	0.21	Abscisic aldehyde	Chlorophyll	Rhizosphere soil
0.6	0.21	Zeatin	Sugarcane weight	Rhizosphere soil
−0.54	0.27	Abscisic aldehyde	Sucrose content	Rhizosphere soil
0.54	0.27	Biotin sulfone	Sugarcane weight	Rhizosphere soil
−0.54	0.27	Abscisic aldehyde	Brix	Rhizosphere soil
−0.54	0.27	Apigenin	Brix	Rhizosphere soil
−0.54	0.27	Sucrose	Brix	Rhizosphere soil
−0.49	0.33	Jasmonic acid	Sucrose content	Rhizosphere soil
0.49	0.33	Jasmonic acid	Sugarcane weight	Rhizosphere soil
0.44	0.38	Phenyllactic acid	Sugarcane weight	Rhizosphere soil
−0.43	0.4	Sucrose	Chlorophyll	Rhizosphere soil
−0.37	0.47	Abscisic acid	Sucrose content	Rhizosphere soil
0.37	0.47	Abscisic acid	Sugarcane weight	Rhizosphere soil

## 4 Discussion

This work aims to illuminate the underpinning mechanism of how specific plant growth and stress-regulating metabolites respond to long-term BC utilization shape soil parameters (e.g., biochemical properties and disease-causing bacteria) and crop growth and unravel the association between these compounds and potential plant growth-promoting bacteria. The adoption of BC as a soil supplement in agriculture has garnered significant traction among many farmers and researchers, primarily due to its eco-friendly role in boosting crop output (Panwar et al., 2019). Graber et al. (2010) documented that BC-treated soil boosted tomato and pepper development and productivity cultivated in fertigated soilless media. A related work also shed light on BC’s role in promoting soil quality and crop growth (Schulz and Glaser, 2012). Here, sugarcane brix, sucrose, and chlorophyll content increased considerably under the BC-supplemented soil. This finding could partly be ascribed to the high presence of zeatin, melatonin, and phenyllactic acid, deemed as plant growth and stress regulators detected in the various plant tissues (Schäfer et al., 2015; Moustafa-Farag et al., 2020), as shown in **Tables 2**–**5**. Furthermore, the ability of BC to act as a slow-release fertilizer and gradually release nutrients, including N, and P (Wang et al., 2022a), as it decays, contributed to this phenomenon. This behavior is consistent with a previous study, where it was revealed that BC-sustained nutrient release provided a steady NH_4_^+^N and NO_3_^-^N supply for crop growth and development (Fallah et al., 2023b), as shown in **Figures 1I–K**. A similar phenomenon was documented in the work of Wang et al. (2022a).

Supplementing soil with BC is thought to be a sustainable soil management strategy that promotes soil biochemical parameters, including soil extracellular enzymes (Burns et al., 2013). BC-treated soil has typically been found to promote soil enzyme activities associated with P and N (Bailey et al., 2011). Here, BC supplemented at the rate of 20 t/hm^2^ significantly promoted soil phosphatase, which is consistent with the results of Liu et al. (2017), where it was reported that phosphatase activities significantly peaked under BC applied at the rate of 20 t/hm^2^. This phenomenon is associated with the crucial role BC utilization plays in decomposing organic matter, which eventually releases organic phosphorus compounds (Wu et al., 2023). However, BC-supplemented soil has also been observed to lower the enzymatic activities tied to ecological processes such as soil C mineralization (Lehmann et al., 2011), primarily due to the high C content in BC (Panwar et al., 2019) or the sorption of enzymes by BC (Czimczik and Masiello, 2007). Here, BC-supplemented soil significantly promoted soil β-glucosidase and cellulase, which slightly agreed with Wang et al.’s (2015a) findings. The increase in these C cycling enzymes could be related to the low availability of a specific nutrient (e.g., soil pH) due to the high increase in C from BC. Additionally, the high pyrolysis temperature (550–650°C) at which the BC used in this study was generated contributed to this phenomenon, as BC derived from such temperature has shown a promising role in promoting C cycling enzymes, evident by the findings of Khadem and Raiesi (2017). We also believe that the potential of BC to adsorb and retain soil nutrients, inhibiting leaching and making them available to soil microorganisms and plants over a protracted period, led to a nutrient-rich environment that promoted the activity of soil microbes and growth, leading to the promotion of soil enzyme activity (Ibrahim et al., 2020). Our finding agrees with a previous work (Jiang et al., 2021), where it was established that BC utilization significantly promoted enzyme activities, including sucrose, phosphatase, and catalase activity.

In this study, soil TN, NH_4_^+^N, and NO_3_^−^N peaked under the BC-supplemented soil, largely due to the highly porous structure and surface area of BC, which allowed it to adsorb and retain soil N compounds. This behavior prevents N leaching and loss, making more N available for crop uptake and microbial activities (Zheng et al., 2013). The peak in these soil nutrients could be associated with several factors. Firstly, the interaction of BC with soil organic matter helps reduce N volatilization (Mandal et al., 2016). Secondly, the ability of BC to enhance the mineralization of soil N (Yoo and Kang, 2012), which eventually increases the soil NO_3_^−^-N, explains the mechanism underpinning this behavior (Wang et al., 2015a). Ultimately, the enhanced microbial activity induced by the application of BC increased soil NO_3_^–^-N and NH_4_^+^-N. This outcome aligns with earlier research findings (Wang et al., 2015a; Fallah et al., 2023c).

Soil amendment practices can induce a shift in soil attributes, including metabolite abundance and composition. Rusli et al. (2022) reported that the total contents of metabolites, including phenolic, anthocyanin, and flavonoid, peaked considerably in the tissues of *Melastoma malabathricum* L. when supplemented with BC. In this work, some potential plant growth and stress-regulating metabolites, including zeatin, melatonin, sucrose, and phenyllactic acid, responded strongly to the BC-supplemented soil. Melatonin is an important phytohormone mediating diverse plant growth processes, including crop growth, yield, seed germination, root elongation, and flowering (Wang et al., 2022b). Teng et al. (2022) reported that melatonin was crucial in promoting plant sucrose and fructose. Moustafa-Farag et al. (2020) also highlighted the decisive aspect of melatonin and its role in mediating environmental stressors. In their work, they explored the significance of melatonin in regulating soil pH and heavy metals. We believe that the ability of BC to alter microbe–plant interactions eventually influenced the secretion of root exudates, thereby triggering the promotion of melatonin, which boosted crop traits and suppressed bacteria pathogens.

Zeatin is regarded as a plant hormone belonging to the cytokinin family and is crucial in regulating different facets of plant growth and development (Schäfer et al., 2015). It has also been documented that zeatin can promote plant nutrient uptake and translocation. For example, trans-zeatin promoted the ability of *Arabidopsis thaliana* plant to fine-tune its shoot growth to adapt to fluctuating environmental conditions (Osugi et al., 2017). Our finding suggests that the high presence of zeatin under the BC-supplemented soil contributed to the increase in crop traits and the promotion of soil nutrients, especially N, NO_3_-N, and NH_4_-N. This behavior corroborates with a previous study (Kawai et al., 2022), in which it was established that trans-zeatin biosynthesis belonging to cytokinin was significantly upregulated in response to the application of N. This finding also aligns with the results documented in the work of Kudo et al. (2012), where cis-zeatin, a type of cytokinin, exhibited a physiological impact on rice growth and development.

Phenyllactic acid is a phenolic compound that can exhibit antimicrobial properties, helping plants cope with oxidative stress triggered by different environmental factors (Kawtharani et al., 2020). This compound also plays crucial roles in plant physiological processes, including signal transduction and regulation of growth and development, as Lavermicocca et al. (2003) documented. These authors proved that phenyllactic acid induced an unpredictable delay in the growth of some mycotoxigenic strains, including *Penicillium citrinum* and *Penicillium verrucosum*. Jiang et al. (2022) recently investigated the performance of phenyllactic acid in inhibiting *Staphylococcus aureus* and found that phenyllactic acid was a potential candidate for controlling *S*. *aureus*. These findings are also consistent with Li et al. (2022) work, implying that the response of this compound to BC contributed to the suppression of disease-causing bacteria. Moreover, the stimulatory effect of BC increased phenyllactic acid through BC–microbe–plant interactions, eventually triggering the secretion of more root exudates, thereby boosting vital soil nutrients and crop traits.

Sucrose plays an important role in plants as an energy source, carbon carrier, storage compound (Ruan, 2014), and involvement in stress responses (Du et al., 2020). In the plant–soil interface, sucrose also contributes to the secretion of root exudates, which promotes beneficial microbial activities (Lopes et al., 2022). Li et al. (2020) established that the peak in sucrose metabolism under BC-supplemented soil promoted microbial functions, eventually boosting soil health and biochemical properties. Likewise, a study showed that the breakdown of sucrose was a key strategy for plant growth improvement (Daloso et al., 2016). In another, Du et al. (2020) highlighted the importance of the different regulation strategies in sucrose allocation, transport, and metabolism during various seed development stages for soybean plants to resist drought stress. We observed that sucrose was significantly upregulated in the stem tissue of the BC-supplemented soil, suggesting that the abundance of sucrose in the stem tissue of the BC-supplemented soil was key in promoting crop growth and microbial community (Gunina and Kuzyakov, 2015).

## 5 Conclusion

Our finding provides valuable information for agronomists, farmers, and environmentalists to make informed decisions about crop production, land use, and soil management practices. Proper soil assessment and understanding of the interaction between the attributes of soil, BC, and metabolites are essential for promoting sustainable agriculture practices and land conservation.

## Data availability statement

We submitted the raw data to the NCBI Sequence Read Archive (accession no. PRJNA929962).

## Author contributions

NF: Conceptualization, Data curation, Formal Analysis, Investigation, Methodology, Validation, Writing – original draft, Writing – review & editing. ZP: Writing – review & editing, Data curation, Investigation, Resources, Software, Validation. ZL: Writing – review & editing, Conceptualization, Project administration, Resources, Software, Supervision. WL: Supervision, Writing – review & editing, Conceptualization, Project administration, Resources. SM: Conceptualization, Data curation, Software, Validation, Writing – review & editing. AA: Resources, Software, Validation, Writing – review & editing. KF: Writing – review & editing. HZ: Conceptualization, Funding acquisition, Project administration, Resources, Supervision, Validation, Visualization, Writing – review & editing.

## References

[B1] Abujabhah I. S. Doyle R. B. Bound S. A. Bowman J. P. (2018). Assessment of bacterial community composition, methanotrophic and nitrogen-cycling bacteria in three soils with different biochar application rates. J. Soils Sediments 18, 148–158. doi: 10.1007/s11368-017-1733-1

[B2] Bailey V. L. Fansler S. J. Smith J. L. Bolton H. Jr (2011). Reconciling apparent variability in effects of biochar amendment on soil enzyme activities by assay optimization. Soil Biol. Biochem. 43, 296–301. doi: 10.1016/j.soilbio.2010.10.014

[B3] Blanchet G. Gavazov K. Bragazza L. Sinaj S. (2016). Responses of soil properties and crop yields to different inorganic and organic amendments in a Swiss conventional farming system. Agric. Ecosyst. Environ. 230, 116–126. doi: 10.1016/j.agee.2016.05.032

[B4] Bulluck R. Brosius M. Evanylo G. Ristaino J. (2002). Organic and synthetic fertility amendment influence soil microbial physical and chemical properties on organic and conventional farms. Appl. Soil Ecol. 19, 147–160. doi: 10.1016/S0929-1393(01)00187-1

[B5] Burns R. G. DeForest J. L. Marxsen J. Sinsabaugh R. L. Stromberger M. E. Wallenstein M. D.. (2013). Soil enzymes in a changing environment: current knowledge and future directions. Soil Biol. Biochem. 58, 216–234. doi: 10.1016/j.soilbio.2012.11.009

[B6] Carpenter S. R. Caraco N. F. Correll D. L. Howarth R. W. Sharpley A. N. Smith V. H. (1998). Nonpoint pollution of surface waters with phosphorus and nitrogen. Ecol. Appl. 8, 559–568. doi: 10.2307/2641247

[B7] Chen W. Gong L. Guo Z. Wang W. Zhang H.-Y. Xianqing L.. (2013). A novel integrated method for large-scale detection, identification, and quantification of widely targeted metabolites: Application in the study of rice metabolomics. Mol. Plant 6, 1769–1780. doi: 10.1093/mp/sst080 23702596

[B8] Chenkun Y. Shuangqian S. Shen Z. Li Y. Mao Y. Zhou J.. (2021). Rice metabolic regulatory network spanning its entire life cycle. Mol. Plant. doi: 10.1016/j.molp.2021.10.005 34715392

[B9] Czimczik C. I. Masiello C. A. (2007). Controls on black carbon storage in soils. Global Biogeochem. Cycles 21, 3. doi: 10.1029/2006GB002798

[B10] Daloso D. M. Williams T. C. R. Antunes W. C. Pinheiro D. P. Müller C. Loureiro M. E.. (2016). Guard cell-specific upregulation of sucrose synthase 3 reveals that the role of sucrose in stomatal function is primarily energetic. New Phytol. 209, 1470–1483. doi: 10.1111/nph.13704 26467445

[B11] Du Y. Zhao Q. Chen L. Yao X. Zhang H. Wu J.. (2020). Effect of drought stress during soybean R2–R6 growth stages on sucrose metabolism in leaf and seed. Int. J. Mol. Sci. 21, 618. doi: 10.3390/ijms21020618 31963537 PMC7013680

[B12] Erb M. Kliebenstein D. (2020). Plant Secondary metabolites as defenses, regulators, and primary metabolites: The blurred functional trichotomy. Plant Physiol. 184, 00433.2020. doi: 10.1104/pp.20.00433 PMC7479915 32636341

[B13] Fallah N. Pang Z. Dong F. Zhou Y. Lin W. Fabrice K. M. A.. (2022). Niche differentiation modulates metabolites abundance and composition in silicon fertilizer amended soil during sugarcane growth. BMC Plant Biol. 22, 1–16. doi: 10.1186/s12870-022-03880-7 36280810 PMC9590199

[B14] Fallah N. Pang S. Lin Z. Nyimbo W. J. Lin W. Mbuya S. N. Fallah N. (2023b). Sustained organic amendments utilization enhances ratoon crop growth and soil quality by enriching beneficial metabolites and suppressing pathogenic bacteria. Front. Plant Sci. 14, 2023. doi: 10.1016/j.geoderma.2018.11.006 PMC10544933 37790789

[B15] Fallah N. Pang Z. Zhang C. Tayyab M. Yang Z. Lin Z.. (2023a). Complementary effects of biochar, secondary metabolites, and bacteria biocontrol agents rejuvenate ratoon sugarcane traits and stimulate soil fertility. Ind. Crops Prod. 202, 117081. doi: 10.1016/j.indcrop.2023

[B16] Fallah N. Tayyab M. Yang Z. Pang Z. Zhang C. Lin Z.. (2023c). Free-living bacteria stimulate sugarcane growth traits and edaphic factors along soil depth gradients under contrasting fertilization. Sci. Rep. 13, 6288. doi: 10.1038/s41598-022-25807-w 37072423 PMC10113235

[B17] Fallah N. Yang Z. Tayyab M. Zhang C. Abubakar A. Lin Z.. (2021). Depth-dependent influence of biochar application on the abundance and community structure of diazotrophic under sugarcane growth. PloS One 16 (7), e0253970. doi: 10.1371/journal.pone.0253970 34280207 PMC8289083

[B18] Ginebra M. Muñoz C. Calvelo-Pereira R. Doussoulin M. Zagal E. (2022). Biochar impacts on soil chemical properties, greenhouse gas emissions and forage productivity: A field experiment. Sci. Total Environ. 806, 150465. doi: 10.1016/j.scitotenv.2021.150465 34582858

[B19] Graber E. R. Harel Y. M. Kolton M. Cytryn E. Silber A. David D. R.. (2010). Biochar impact on development and productivity of pepper and tomato grown in fertigated soilless media. Plant Soil 337, 481–496. doi: 10.1007/s11104-010-0544-6

[B20] Gul S. Whalen J. K. Thomas B. W. Sachdeva V. Deng H. (2015). Physico-chemical properties and microbial responses in biochar-amended soils: mechanisms and future directions. Agric. Ecosyst. Environ. 206, 46–59. doi: 10.1016/j.agee.2015.03.015

[B21] Gunina A. Kuzyakov Y. (2015). Sugars in soil and sweets for microorganisms: review of origin, content, composition and fate. Soil Biol. Biochem. 90, 87–100. doi: 10.1016/j.soilbio.2015.07.021

[B22] Hartmann T. (2007). From waste products to ecochemicals: fifty years research of plant secondary metabolism. Phytochemistry 68, 2831–2846. doi: 10.1016/j.phytochem.2007.09.017 17980895

[B23] Hu L. Wu Z. Robert C. A. M. Ouyang X. Züst T. Mestrot A.. (2021). Soil chemistry determines whether defensive plant secondary metabolites promote or suppress herbivore growth. Proc. Natl. Acad. Sci. 118, e2109602118. doi: 10.1101/2021.05.14.444261 34675080 PMC8639379

[B24] Ibrahim M. Hu K. Tong C. Xing S. Zou S. Mao Y. (2020). De-ashed biochar enhances nitrogen retention in manured soil and changes soil microbial dynamics. Geoderma 278, 114589. doi: 10.1016/j.geoderma.2020.114589

[B25] Jiang Y. Wang X. Zhao Y. Zhang C. Jin Z. Shan S.. (2021). Effects of biochar application on enzyme activities in tea garden soil. Front. Bioeng. Biotechnol. 9. doi: 10.3389/fbioe.2021.728530 PMC8490741 34621730

[B26] Jiang Y.-H. Ying J.-P. Xin W.-G. Yang L.-Y. Li X.-Z. Zhang Q.-L. (2022). Antibacterial activity and action target of phenyllactic acid against Staphylococcus aureus and its application in skim milk and cheese. J. Dairy Sci. 105, 9463–9475. doi: 10.3168/jds.2022-22262 36270872

[B27] Jindo K. Mizumoto H. Sawada Y. Sanchez-Monedero M. A. Sonoki T. (2014). Physical and chemical characterization of biochars derived from different agricultural residues. Biogeosciences 11, 6613–6621. doi: 10.5194/bgd-11-11727-2014

[B28] Junaid Rao M. Duan M. Yang M. Li M. Wang L. (2022). Sugarcane rind secondary metabolites and their antioxidant activities in eleven cultivated sugarcane varieties. Sugar Tech 24, 6. doi: 10.1007/s12355-021-01097-w

[B29] Kawai M. Tabata R. Ohashi M. Honda H. Kamiya T. Kojima M.. (2022). Regulation of ammonium acquisition and use in *Oryza longistaminata* ramets under nitrogen source heterogeneity. Plant Physiol. 188, 2364–2376. doi: 10.1093/plphys/kiac025 35134987 PMC8968255

[B30] Kawtharani H. Snini S. P. Heang S. Bouajila J. Taillandier P. Mathieu F.. (2020). Phenyllactic acid produced by *Geotrichum Candidum* reduces *Fusarium sporotrichioides* and *F. langsethiae* growth and T-2 toxin concentration. Toxins (Basel). 12 (4), 209. doi: 10.3390/toxins12040209 32224845 PMC7232515

[B31] Khadem A. Raiesi F. (2017). Influence of biochar on potential enzyme activities in two calcareous soils of contrasting texture. Geoderma. 308, 149–158. doi: 10.1016/j.geoderma.2017.08.004

[B32] Kudo T. Makita N. Kojima M. Tokunaga H. Sakakibara H. (2012). Cytokinin activity of cis-zeatin and phenotypic alterations induced by overexpression of putative cis-Zeatin-O-glucosyltransferase in rice. Plant Physiol. 160, 319–331. doi: 10.1104/pp.112.196733 22811434 PMC3440209

[B33] Lavermicocca P. Francesca V. Angelo V. (2003). Antifungal activity of phenyllactic acid against molds isolated from bakery products. Appl. Environ. Microbiol. 69, 634–640. doi: 10.1128/AEM.69.1.634-640.2003 12514051 PMC152452

[B34] Lehmann J. Rillig M. C. Thies J. Masiello C. A. Hockaday W. C. Crowley D. (2011). Biochar effects on soil biota–a review. Geoderma. 43, 1812–1836. doi: 10.1016/j.soilbio.2011.04.022

[B35] Li X. Yao S. Bian Y. Jiang X. Song Y. (2020). The combination of biochar and plant roots improves soil bacterial adaptation to PAH stress: Insights from soil enzymes, microbiome, and metabolome. J. Hazard. Mater. 400, 123227. doi: 10.1016/j.jhazmat.2020.123227 32585520

[B36] Li M. Yao B. Meng X. (2022). Inhibitory effect and possible mechanism of phenyllactic acid on *Aspergillus flavus* spore germination. J. Basic Microbiol. 62, 1457–1466. doi: 10.1002/jobm.202200274 35925551

[B37] Liu S. Meng J. Jiang L. Yang X. Lan Y. Cheng X.. (2017). Rice husk biochar impacts soil phosphorous availability, phosphatase activities and bacterial community characteristics in three different soil types. Appl. Soil Ecol. 116, 12–22. doi: 10.1016/j.apsoil.2017.03.020

[B38] Liu Z. Zhou H. Xie W. Yang Z. Lv Q. (2021). Long-term effects of maize straw return and manure on the microbial community in cinnamon soil in Northern China using 16S rRNA sequencing. PloS One 16, e0249884. doi: 10.1371/journal.pone.0249884 33886593 PMC8062091

[B39] Lopes L. D. Wang P. Futrell S. L. Schachtman D. P. (2022). Sugars and jasmonic acid concentration in root exudates affect maize rhizosphere bacterial communities. Appl. Environ. Microbiol. 88, e00971–e00922. doi: 10.1128/aem.00971-22 36073926 PMC9499034

[B40] Luan H. Gao W. Huang S. Tang J. Li M. Zhang H.. (2020). Substitution of manure for chemical fertilizer affects soil microbial community diversity, structure and function in greenhouse vegetable production systems. PloS One 15, e0214041. doi: 10.1371/journal.pone.0214041 32084129 PMC7034837

[B41] Mandal S. Thangarajan R. Bolan N. S. Sarkar B. Khan N. Ok Y. S.. (2016). Biochar-induced concomitant decrease in ammonia volatilization and increase in nitrogen use efficiency by wheat. Chemosphere 142, 120–127. doi: 10.1016/j.chemosphere.2015.04.086 25959224

[B42] Martí E. Sierra J. Domene X. Mumbrú M. Cruañas R. Garau M. A. (2021). One-year monitoring of nitrogen forms after the application of various types of biochar on different soils. Geoderma 402, 115178. doi: 10.1016/j.geoderma.2021.115178

[B43] Mithöfer A. Boland W. (2012). Plant defense against herbivores: chemical aspects. Annu. Rev. Plant Biol. 63, 431–450. doi: 10.1146/annurev-arplant-042110-103854 22404468

[B44] Moustafa-Farag M. Elkelish A. Dafea M. Khan M. Arnao M. B. Abdelhamid M. T.. (2020). Role of melatonin in plant tolerance to soil stressors: Salinity, pH and heavy metals. Molecules 25, 5359. doi: 10.3390/molecules25225359 33212772 PMC7696660

[B45] Osugi A. Kojima M. Takebayashi Y. Ueda N. Kiba T. Sakakibara H. (2017). Systemic transport of trans-zeatin and its precursor have differing roles in *Arabidopsis* shoots. Nat. Plants 3, 8. doi: 10.1038/nplants.2017.112. nplants2017112. 28737742

[B46] Panchal P. Preece C. Penuelas J. Giri J. (2022). Soil carbon sequestration by root exudates. Trends Plant Sci. 27, 749–757. doi: 10.1016/j.tplants.2022.04.009 35606255

[B47] Pang Z. Chen J. Wang T. Gao C. Li Z. Guo L.. (2021). Linking plant secondary metabolites and plant microbiomes: A review. Front. Plant Sci. 12. doi: 10.3389/fpls.2021.621276 PMC7961088 33737943

[B48] Pang Z. Huang J. Fallah N. Lin W. Yuan Z. Hu C. (2022). Combining N fertilization with biochar affects root-shoot growth, rhizosphere soil properties and bacterial communities under sugarcane monocropping. Ind. Crops Prod. 182, 114899. doi: 10.1016/j.indcrop.2022.114899

[B49] Panwar N. L. Pawar A. Salvi B. L. (2019). Comprehensive review on production and utilization of biochar. SN Appl. Sci. 1, 168. doi: 10.1007/s42452-019-0172-6

[B50] Popp J. Pető K. Nagy J. (2013). Pesticide productivity and food security. A review. Agron. Sustain. Dev. 33, 243–255. doi: 10.1007/s13593-012-0105-x

[B51] Ramlow M. Foster E. J. Del Grosso S. J. Cotrufo M. F. (2019). Broadcast woody biochar provides limited benefits to deficit irrigation maize in Colorado. Agric. Ecosyst. Environ. 269, 71–81. doi: 10.1016/j.agee.2018.09.017

[B52] Ruan Y.-L. (2014). Sucrose metabolism: Gateway to Diverse carbon use and sugar signaling. Annu. Rev. Plant Biol. 65, 1. doi: 10.1146/annurev-arplant-050213-040251 24579990

[B53] Rusli L. Rosazlin A. Yaacob J. S. Osman N. (2022). Organic amendments effects on nutrient uptake, secondary metabolites, and antioxidant properties of *Melastoma malabathricum* L. Plants 11, 153. doi: 10.3390/plants11020153 35050041 PMC8778759

[B54] Schäfer M. Brütting C. Meza-Canales I. D. Großkinsky D. K. Vankova R. Baldwin I. T.. (2015). The role of cis-zeatin-type cytokinins in plant growth regulation and mediating responses to environmental interactions. J. Exp. Bot. 66, 4873–4884. doi: 10.1093/jxb/erv214 25998904 PMC5147713

[B55] Schärer M.-L. Dietrich L. Kundel D. Mäder P. Kahmen A. (2022). Reduced plant water use can explain higher soil moisture in organic compared to conventional farming systems. Agric. Ecosyst. Environ. 332, 107915. doi: 10.1016/j.agee.2022.107915

[B56] Schulz H. Glaser B. (2012). Effects of biochar compared to organic and inorganic fertilizers on soil quality and plant growth in a greenhouse experiment. J. Plant Nutr. Soil Sci. 175, 410–422. doi: 10.1002/jpln.201100143

[B57] Sun J. Zhang Q. Zhou J. Wei Q. (2014). Pyrosequencing technology reveals the impact of different manure doses on the bacterial community in apple rhizosphere soil. Appl. Soil Ecol. 78, 28–36. doi: 10.1016/j.apsoil.2014.02.004

[B58] Tan Y. Cui Y. Li H. Kuang A. Li X. Wei Y.. (2017). Rhizospheric soil and root endogenous fungal diversity and composition in response to continuous Panax notoginseng cropping practices. Microbiol. Res. 194, 10–19. doi: 10.1016/j.micres.2016.09.009 27938858

[B59] Tang X. He Y. Zhang Z. Wu H. He L. Jiang J.. (2022). Beneficial shift of rhizosphere soil nutrients and metabolites under a sugarcane/peanut intercropping system. Front. Plant Sci. 13. doi: 10.3389/fpls.2022.1018727 PMC9757493 36531399

[B60] Teng Z. Zheng W. Jiang S. Hong S.-B. Zhu Z. Zang Y. (2022). Role of melatonin in promoting plant growth by regulating carbon assimilation and ATP accumulation. Plant Sci. 319, 111276. doi: 10.1016/j.plantsci.2022.111276 35487649

[B61] Toju H. Yamamoto S. Tanabe A. S. Hayakawa T. Ishii H. S. (2016). Network modules and hubs in plant-root fungal biomes. J. R. Soc Interface 13, 20151097. doi: 10.1098/rsif.2015.1097 26962029 PMC4843674

[B62] Van-Dijk M. Morley T. Rau M. L. Saghai Y. (2021). A meta-analysis of projected global food demand and population at risk of hunger for the period 2010–2050. Nat. Food 2, 494–501. doi: 10.1038/s43016-021-00322-9 37117684

[B63] Wang Q. Garrity G. M. Tiedje J. M. Cole J. R. (2007). Naive Bayesian classifier for rapid assignment of rRNA sequences into the new bacterial taxonomy. Appl. Environ. Microbiol. 73, 5261–5267. doi: 10.1128/AEM.00062-07 17586664 PMC1950982

[B64] Wang C. Luo D. Zhang X. Huang R. Cao Y. Liu G.. (2022a). Biochar-based slow-release of fertilizers for sustainable agriculture: A mini review. Environ. Sci. Ecotechnology 10, 100167. doi: 10.1016/j.ese.2022.100167 PMC9488105 36159737

[B65] Wang K. Xing Q. Ahammed G. J. Zhou J. (2022b). Functions and prospects of melatonin in plant growth, yield, and quality. J. Exp. Bot. 73, 5928–5946. doi: 10.1093/jxb/erac233 35640564

[B66] Wang X. Zhou W. Liang G. Song D. Zhang X. (2015a). Characteristics of maize biochar with different pyrolysis temperatures and its effects on organic carbon, nitrogen and enzymatic activities after addition to fluvo-aquic soil. Sci. Total Environ. 538, 137–144. doi: 10.1016/j.scitotenv.2015.08.026 26298256

[B67] Wang Z. Zong H. Zheng H. Liu G. Chen L. Xing B. (2015b). Reduced nitrification and abundance of ammonia-oxidizing bacteria in acidic soil amended with biochar. Chemosphere 138, 576–583. doi: 10.1016/j.chemosphere.2015.06.084 26210022

[B68] Watson C. A. Atkinson D. Gosling P. Jackson L. R. Rayns F. W. (2002). Managing soil fertility in organic farming systems. Soil Use Manage. 18, 239–247. doi: 10.1111/j.1475-2743.2002.tb00265.x

[B69] Wen T. Yu G.-H. Hong W.-D. Yuan J. Niu G.-Q. Xie P.-H.. (2022). Root exudate chemistry affects soil carbon mobilization via microbial community reassembly. Fundam. Res. 2, 697–707. doi: 10.1016/j.fmre.2021.12.016 PMC11197519 38933120

[B70] Woolf D. Amonette J. E. Street-Perrott F. A. Lehmann J. Joseph S. (2010). Sustainable biochar to mitigate global climate change. Nat. Commun. 1, 56. doi: 10.1038/ncomms1053 20975722 PMC2964457

[B71] Wu Q. Zhang J. Liu X. Chang T. Wang Q. Shaghaleh H.. (2023). Effects of biochar and vermicompost on microorganisms and enzymatic activities in greenhouse soil. Front. Environ. Sci. 10. doi: 10.3389/fenvs.2022.1060277

[B72] Yang X. Sun Q. Yuan J. Fu S. Lan Y. Jiang X.. (2022). Successive corn stover and biochar applications mitigate NO emissions by altering soil physicochemical properties and N-cycling-related enzyme activities: A five-year field study in Northeast China. Agric. Ecosyst. Environ. 340, 108183. doi: 10.1016/j.agee.2022.108183

[B73] Yoo G. Kang H. (2012). Effects of biochar addition on greenhouse gas emissions and microbial responses in a short-term laboratory experiment. J. Environ. Qual. 41, 1193–1202. doi: 10.2134/jeq2011.0157 22751062

[B74] Yuan Z. Dong F. Pang Z. Fallah N. Zhou M. Y. Li Z.. (2022). Integrated metabolomics and transcriptome analyses unveil pathways involved in sugar content and rind color of two sugarcane varieties. Front. Plant Sci. 1912. doi: 10.3389/fpls.2022.921536 PMC9244704 35783968

[B75] Zhao Z.-B. He J.-Z. Geisen S. Han L.-L. Wang J.-T. Shen J.-P.. (2019). Protist communities are more sensitive to nitrogen fertilization than other microorganisms in diverse agricultural soils. Microbiome 7, 1–16. doi: 10.1186/s40168-019-0647-0 30813951 PMC6393985

[B76] Zheng H. Wang Z. Deng X. Herbert S. Xing B. (2013). Impacts of adding biochar on nitrogen retention and bioavailability in agricultural soil. Geoderma 206, 32–39. doi: 10.1016/j.geoderma.2013.04.018

