# Supplementary material for: Plant growth and stress-regulating metabolite response to biochar utilization boost crop traits and soil health
Source: Front Plant Sci. 2023 Oct 12;14:1271490. doi: 10.3389/fpls.2023.1271490 (PMC10600501; doi:10.3389/fpls.2023.1271490)
Supplement: Supplementary file 1 [file DataSheet_1.docx]

**Expression of metabolites detected in the various in the different plant compartments**


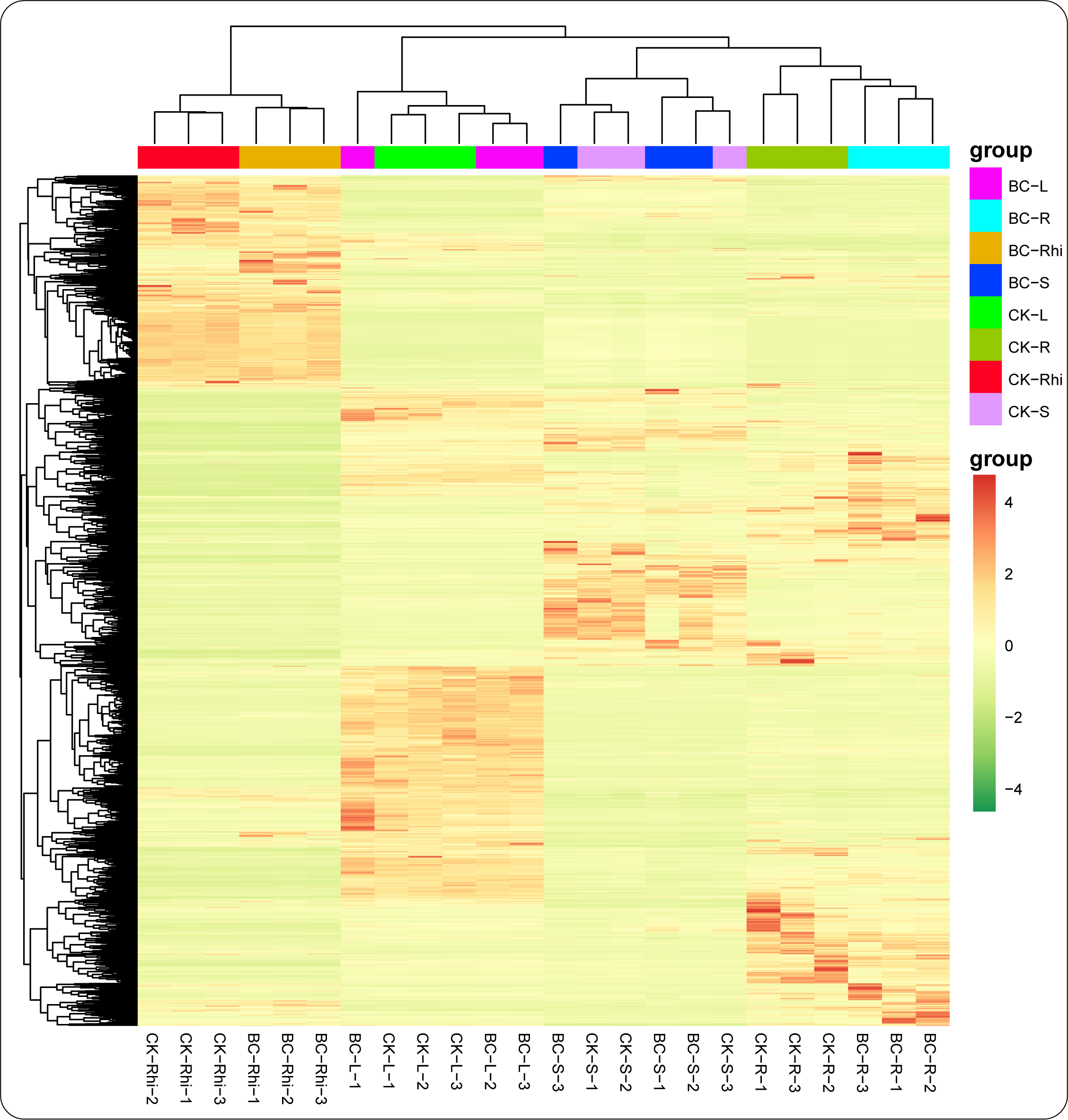


**Figure S1.** Heatmap illustrating the expression patterns of a specific group of metabolite abundance in the various compartments under the BC-supplemented soil and the CK treatment.

**Differentially abundant and enriched pathways metabolites in the different compartments of the crop**


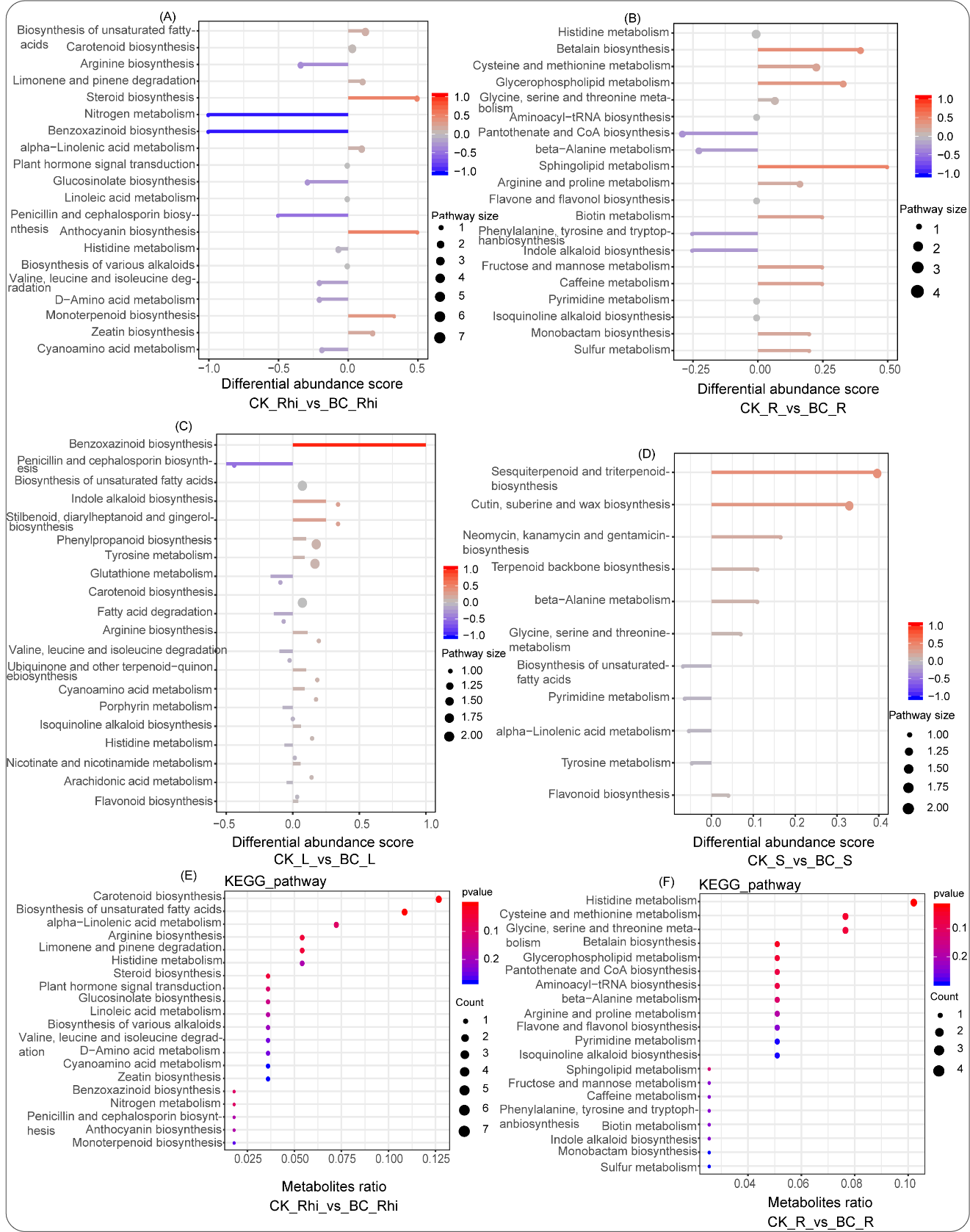


**Figure S2.** Differentially abundance of metabolites function in the rhizosphere soil (A), root tissue (B), leaf tissue (C), and stem tissue (D) of the BC-supplemented field compared with the CK treatment. Enriched KEGG signaling pathway of metabolites in the rhizosphere soil (E) and root tissue (F) under the BC-supplemented field compared with the CK treatment.

**Bacteria abundance and community in plant compartments response to biochar-supplemented soil**


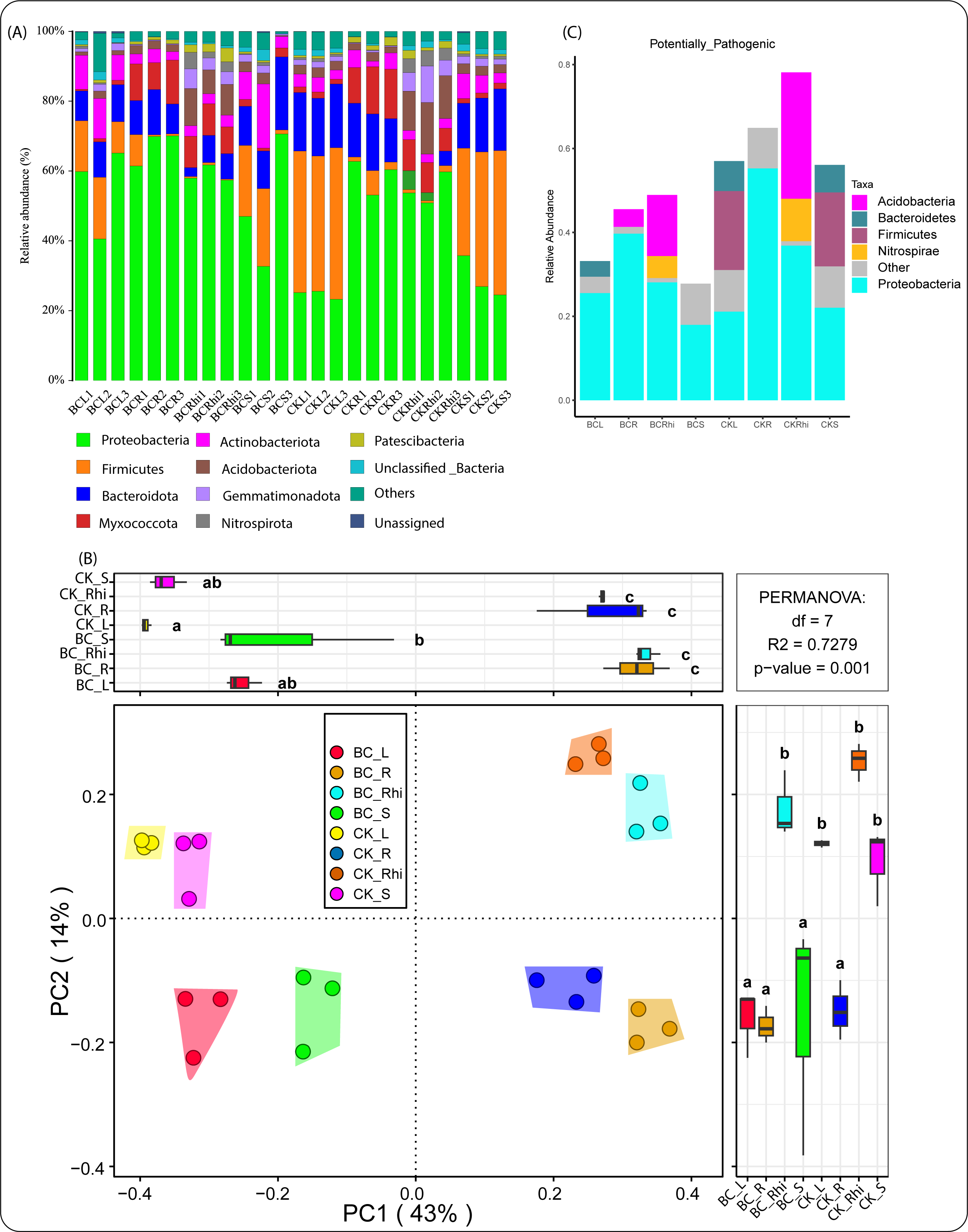


**Figure. S3.** Bacterial relative abundance in the different compartments under both treatments (A). Principal component analysis (PCA) of the entire bacteria detected in the samples (B). BugBase functional analysis illustrating bacterial pathogen detected in the different compartments of the crop under both treatments (C).
